# Crisis in the Chest: Acute Chest Syndrome as a Sequela of Tricuspid Valve Endocarditis

**DOI:** 10.7759/cureus.61061

**Published:** 2024-05-25

**Authors:** Nishal N Patel, Ugochukwu Ebubechukwu, Sunil E Saith, Onyinye S Ugoala, Alexa B Kahn, Samy I McFarlane, Sabu John

**Affiliations:** 1 Internal Medicine, State University of New York Downstate Health Sciences University, Brooklyn, USA; 2 Cardiology, State University of New York Downstate Medical Center, Brooklyn, USA; 3 Internal Medicine, Texas Tech University Health Sciences Center, Amarillo, USA; 4 Cardiology, Brookdale University Hospital and Medical Center, Brooklyn, USA; 5 Cardiology, State University of New York Downstate Health Sciences University, Brooklyn, USA

**Keywords:** vaso-occlusive crisis, infective endocarditis, septic emboli, septic pulmonary emboli, sickle cell crisis, tricuspid valve endocarditis, acute chest syndrome (acs)

## Abstract

The management of acute chest syndrome (ACS) in sickle cell disease occurring concurrently with pulmonary embolism resulting from tricuspid valve endocarditis poses an atypical challenge. We present a case in which this complex interaction occurs and the prompt interventions that were utilized to give the best possible outcome.

## Introduction

Acute chest syndrome (ACS) is a well-recognized and potentially life-threatening complication associated with sickle cell disease. It is characterized by a constellation of symptoms, including chest pain, dyspnea, and fever, often leading to significant morbidity and mortality [1]. There has been a growing recognition of infectious endocarditis as a precipitating factor for ACS. Endocarditis, characterized by inflammation of the endocardium, often involves the heart valves and can lead to the embolization of infected material to various organs, including the lungs, brain, and kidneys. Imaging is pertinent in the diagnosis of both disorders, including computed tomography (CT) angiography and echocardiogram. The coalescence of these two pathologies can result in a clinical picture that intricately combines the complications of sepsis with the pulmonary compromise seen in ACS. In this study, we present a case of tricuspid valve endocarditis precipitating septic pulmonary emboli and acute chest syndrome.

## Case presentation

Investigations

A 35-year-old male with a history of sickle cell disease presents with chest pain, difficulty breathing, and diffuse body aches of three days duration. On examination, the patient was lethargic, in respiratory distress, afebrile, and in acute pain. He was hypotensive with a blood pressure of 70/45 mmHg, tachycardic with a heart rate of 109 beats/minute, tachypneic, and saturating at 94% on room air. A cardiac examination revealed a new systolic murmur over the left sternal border. He was also noted to have a left jugular central venous catheter (CVC), scattered excoriation marks over the left upper extremities, bilateral pitting pedal edema, and deep scleral icterus.

Diagnosis

**Figure 1:**
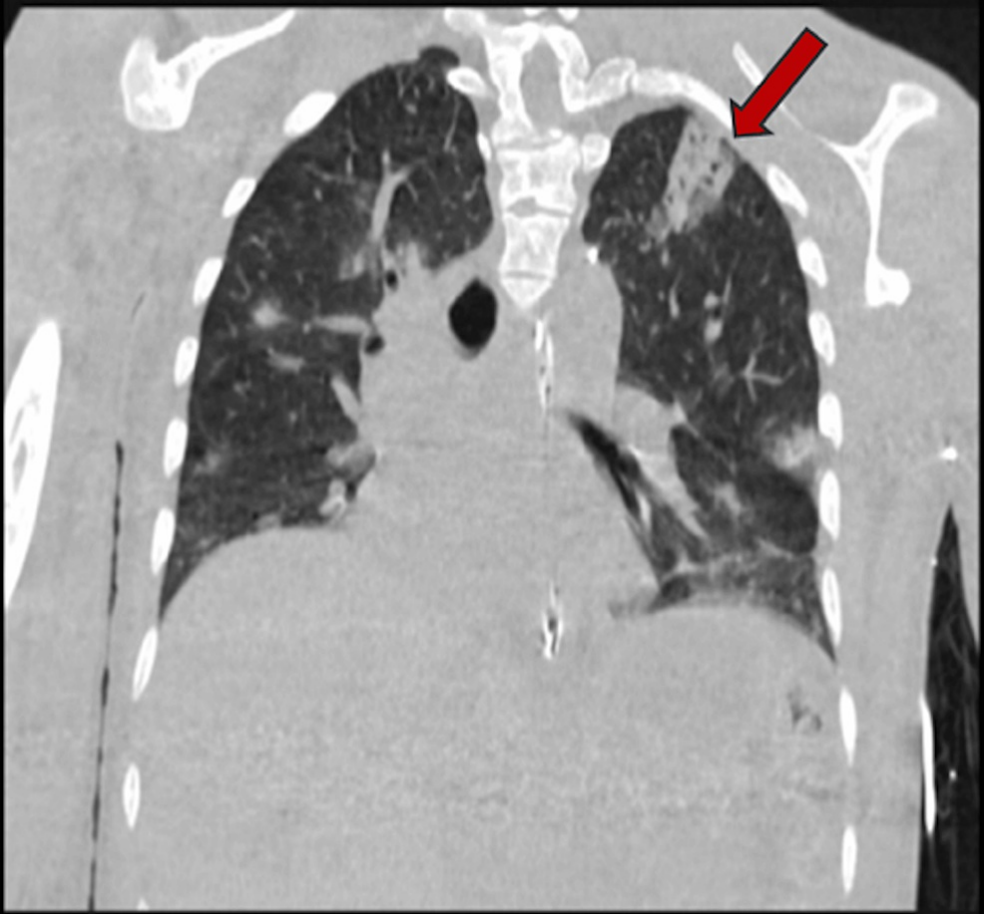
Wedge-shaped ground glass lesions with bubbly central cavitations in the right upper lobe of the lung identified by the red arrow

**Figure 2:**
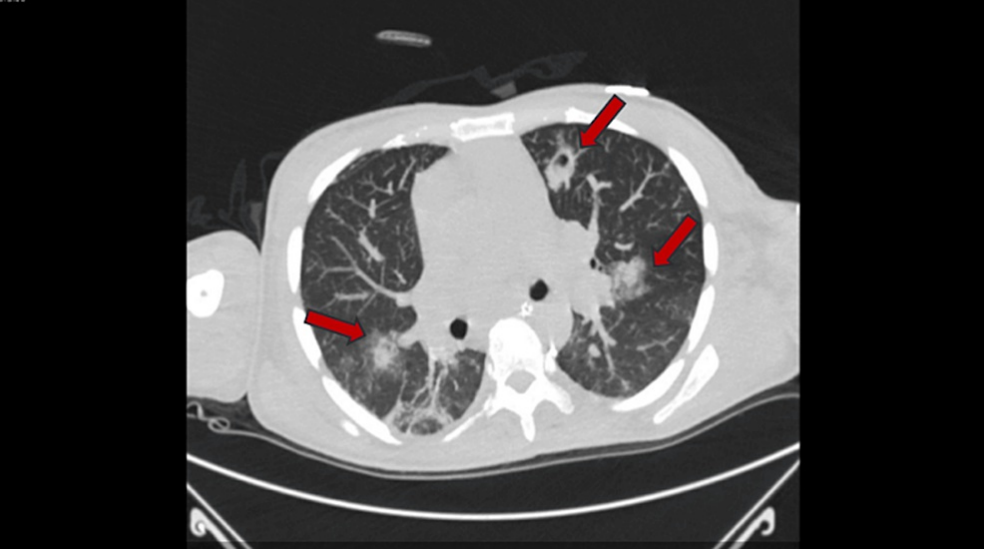
Diffusely distributed ground glass nodules identified by red arrows

**Figure 3:**
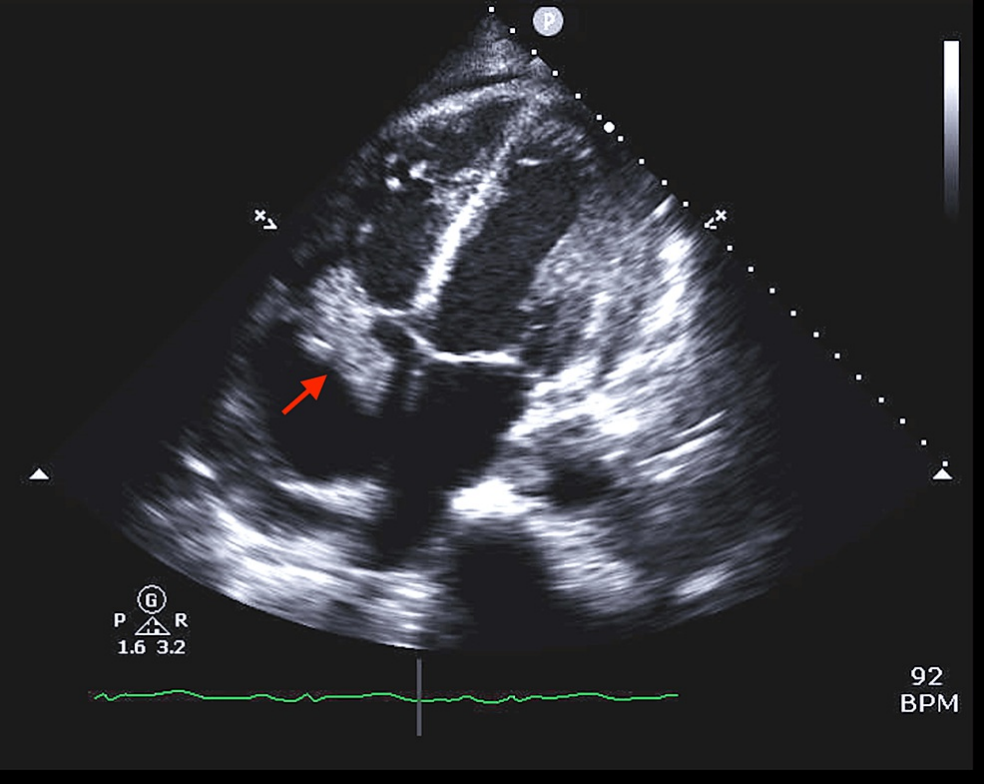
Vegetation on the posterior leaflet of the tricuspid valve measuring 4.2 cm × 2.3 cm identified by the red arrow

Laboratory testing was remarkable for a markedly elevated white blood cell count with neutrophil predominance, decreased hemoglobin and platelet count, elevated reticulocyte count, decreased renal function, lactic acidosis, and elevated bilirubin (Table 1). Urinalysis was positive for dark-colored urine and bilirubin. While pending culture results, gram staining showed gram-positive cocci in clusters. Electrocardiogram (EKG) showed a prolonged QT interval. Chest X-ray revealed a right lower lung and left midlung/upper lung ill-defined opacities suspicious for pneumonia versus acute chest syndrome. Renal and right upper quadrant ultrasound were unremarkable. CT of the chest without contrast showed diffusely distributed ground glass nodules with central cavitation, feeding vessels highly suspicious for septic pulmonary emboli, and wedge-shaped ground glass lesions with bubbly central components in the anterior right lung apex and left lung apex, which may reflect areas of pulmonary infarction and necrosis (Figure 1 and Figure 2). Transthoracic echocardiography revealed a large (4.2 cm × 2.3 cm), highly mobile, multilobular mass on the posterior leaflet of the tricuspid valve consistent with large tricuspid vegetation with severe tricuspid regurgitation and an echogenic mass (about 0.7 cm × 0.56 cm) on the ventricular side of the pulmonic valve (Figure 3). Blood cultures returned positive for methicillin-susceptible *Staphylococcus aureus* after 24 hours.

**Table 1:** Laboratory values on initial presentation WBC: white blood cell, RBC: red blood cell, BUN: blood urea nitrogen, Cr: creatinine, LDH: lactate dehydrogenase

Parameter	Value	Normal range
WBC count	60,840 cells/µL	4,000-11,000 cells/µL
Neutrophil	94%	40%-75%
Hemoglobin	4.6 g/dL	13.8-17.2 g/dL (male), 12.1-15.1 g/dL (female)
RBC	1.59 cells/µL	4.5-6 cells/µL (male), 4-5.2 cells/µL (female)
Reticulocyte count	34.20%	0.5%-2.5%
Platelet count	32,000 cells/µL	150,000-450,000 cells/µL
Lactic acid	5 mmol/L	0.5-2.2 mmol/L
BUN	80 mg/dL	7-20 mg/dL
Cr	4.3 mg/dL	0.6-1.2 mg/dL
LDH	489 U/L	140-280 U/L
Total bilirubin	15.1 mg/dL	0.3-1 mg/dL
Direct bilirubin	12.57 mg/dL	0.1-0.3 mg/dL

Treatment

Given the patient's clinical, laboratory, and initial imaging findings, he was started on gentle intravenous hydration to avoid fluid overload and analgesics. The patient's CVC was removed, and he was empirically started on vancomycin and piperacillin-tazobactam for possible acute chest syndrome. Additionally, hematology was consulted for exchange blood transfusion. A repeat complete blood count (CBC) showed a hemoglobin of 3.2 g/dL suggestive of ongoing hemolysis, and he was transfused with three units of packed red blood cells and a unit of platelets. He was intubated and admitted to the medical intensive care unit for severe respiratory acidosis and started on vasopressors for hypotension. After reviewing the sensitivity results, vancomycin and piperacillin were discontinued, and treatment was initiated with nafcillin. The patient had an exchange blood transfusion the next day and was continued on analgesics, antibiotics, and other supportive care. Further workup for possible cause of markedly elevated white blood cell count included a lymph node biopsy, which was negative for lymphoma. Due to the persistence of elevated white blood cells in the setting of a large mobile tricuspid vegetation, cardiothoracic surgery was consulted for tricuspid valve replacement.

Follow-up and outcomes

The patient was assessed by cardiothoracic surgery and deemed to be a good candidate for valve replacement. The patient underwent a successful tricuspid valve replacement and was continued on intravenous antibiotics for eight weeks. He was subsequently extubated, alert, awake, and interactive. On follow-up, the patient appeared well with no apparent signs of distress. No organisms were detected in repeat blood cultures. The patient stated satisfaction with the overall management and treatment outcome.

## Discussion

The interplay between infective endocarditis (IE) and pulmonary involvement manifests notably in the formation of septic emboli, which adds complexity to the management of sickle cell patients presenting with ACS. Pulmonary complications in endocarditis affect approximately 7% of patients, but among intravenous drug users with infective endocarditis, the incidence rises significantly to 75% [2,3]. The patient presented did not have a history of intravenous drug abuse but does have an extensive history of vaso-occlusive crisis and ACS requiring long-term central venous catheter placement. Intravascular devices, cardiac implantable electronic devices, and underlying right-sided cardiac anomalies are additional risk factors for right-sided IE. Central venous catheters are beneficial in sickle cell patients requiring frequent transfusion but pose a high risk for infection associated with sepsis and thrombosis.

Infective endocarditis could play a role in inciting rapidly progressive acute chest syndrome, defined as respiratory failure ≤ 24 hours after the onset of respiratory symptoms. This presentation occurs more frequently in adults and is present in up to one-fifth of adults with a history of ACS [4]. A retrospective cohort study by Chaturvedi et al. [4] found that the sole predictor of developing rapidly progressive ACS was a decline in platelet count at presentation, which was seen in the patient. In the study, mortality was 6% in patients with rapidly progressive acute chest versus 0% in patients without. Identifying this decline in platelet count could aid in evaluating ACS severity.

The management of ACS resulting from septic pulmonary emboli in endocarditis requires a dual approach. Rapid initiation of targeted antibiotic therapy is crucial not only for treating endocarditis but also for addressing the systemic effects of sepsis. Exchange transfusion is utilized to enhance oxygenation and decrease sickling in patients. Additionally, pain control and respiratory support, either by non-invasive or invasive mechanical ventilation in case of severe respiratory failure, is often needed until the crisis subsides [5].

Timely surgical intervention, when indicated, is vital to prevent complications and enhance outcomes in native valve endocarditis. A meta-analysis by Narayanan et al. [6] found that in patients who had surgical intervention at seven days or less, the odds ratio (OR) of all-cause mortality was 0.61 (95% confidence interval (CI): 0.39 to 0.96, p=0.034), and in those who had surgical intervention within 8-20 days, the OR of mortality was 0.64 (95% CI: 0.48 to 0.86, p=0.003) compared with conservative management. The European Society of Cardiology (ESC) guidelines recommend surgery for right-sided IE in patients receiving appropriate antibiotic therapy who have acute severe tricuspid regurgitation resulting in right ventricular dysfunction, recurrent pulmonary emboli requiring ventilatory support or with large (>20 mm) residual tricuspid vegetations, or involvement of left-sided structures [7,8].

## Conclusions

This case report underscores the intricate interplay between infective endocarditis and acute chest syndrome in patients with sickle cell disease. Timely initiation of appropriate antibiotic therapy is essential for addressing both endocarditis and sepsis. Additionally, supportive measures such as exchange transfusion and respiratory support play crucial roles in managing ACS. Early surgical intervention, guided by clinical indications and guidelines, is paramount in preventing complications and improving outcomes in cases of native valve endocarditis. A multidisciplinary team incorporating cardiology, hematology, infectious disease, critical care, and cardiothoracic surgery is crucial in optimizing patient care.
